# Computational analyses of ancient pathogen DNA from herbarium samples: challenges and prospects

**DOI:** 10.3389/fpls.2015.00771

**Published:** 2015-09-24

**Authors:** Kentaro Yoshida, Eriko Sasaki, Sophien Kamoun

**Affiliations:** ^1^Laboratory of Plant Genetics, Graduate School of Agricultural Science, Kobe UniversityKobe, Japan; ^2^The Sainsbury Laboratory, Norwich Research ParkNorwich, UK; ^3^Gregor Mendel Institute, Austrian Academy of Sciences, ViennaAustria

**Keywords:** herbaria, pathogenomics, *Phytophthora infestans*, plant–pathogen interaction, linkage, haplotype

## Abstract

The application of DNA sequencing technology to the study of ancient DNA has enabled the reconstruction of past epidemics from genomes of historically important plant-associated microbes. Recently, the genome sequences of the potato late blight pathogen *Phytophthora infestans* were analyzed from 19th century herbarium specimens. These herbarium samples originated from infected potatoes collected during and after the Irish potato famine. Herbaria have therefore great potential to help elucidate past epidemics of crops, date the emergence of pathogens, and inform about past pathogen population dynamics. DNA preservation in herbarium samples was unexpectedly good, raising the possibility of a whole new research area in plant and microbial genomics. However, the recovered DNA can be extremely fragmented resulting in specific challenges in reconstructing genome sequences. Here we review some of the challenges in computational analyses of ancient DNA from herbarium samples. We also applied the recently developed linkage method to haplotype reconstruction of diploid or polyploid genomes from fragmented ancient DNA.

One recent application of ancient DNA sequencing to plant pathology focused on whole genome sequencing of the potato late blight pathogen *Phytophthora* *infestans* from herbarium specimens of infected potato leaves (Martin et al., 2013; Yoshida et al., 2013). Some of these samples were collected during the Irish potato famine in the 19th century, a dramatic historic event that was triggered by *P. infestans*. Comparisons between genome sequences of modern and historic samples revealed that the 19th century pandemic was caused by the clonal lineage HERB-1, which is related to the US-1 clonal lineage that became dominant in the 20th century (Yoshida et al., 2013, 2014; Martin et al., 2014). HERB-1 carries a particular set of effector genes, which is different from the 20th and 21st century isolates, and indicate that this clonal lineage had a distinct pattern of virulence (Yoshida et al., 2013). In this article, we discuss some of the problems that arose during the analysis of the genome sequence data obtained from herbarium material (Yoshida et al., 2013). In particular, we discuss the challenge of haplotype reconstruction, and introduce haplotype reconstruction of effector genes using a linkage method.

## The Physical Characteristics of Ancient DNA in Herbarium Samples

DNA is well preserved in herbarium samples but is low in quantity and fragmented into small pieces (Martin et al., 2013; Yoshida et al., 2013). To avoid DNA contamination from modern isolates, treatment of ancient DNA should be undertaken in sterile conditions in a laboratory with no prior exposure to the pathogens of interest in the herbarium samples. However, the physical state of ancient DNA is appropriate for next generation sequencing that can generate massive numbers of short sequences that match the size of the preserved fragments (<100 bp). Also, given that DNA in herbarium samples is already broken into pieces, physical or enzymatic fragmentation that is normally performed during library preparation can be skipped. In the two studies of the 19th century *P. infestans*-infected potato leaves, the mean or median fragment length ranged from about 50 to 86 bp (Martin et al., 2013; Yoshida et al., 2013). Full fragment sequences could be retrieved with 100 bp single-end DNA sequencing, but 100 bp paired-end sequencing would be recommended in order to produce reads with higher quality. Higher error rates in both paired reads are known to occur with the Illumina platform. By merging paired-end reads to a single sequence, the overlapping regions can be used for correcting these sequencing errors. On the other hand, DNA fragmentation impedes long-read sequencing and creates difficulties in reconstructing haplotypes. There is a need for bioinformatics tools to compensate for this shortcoming when analyzing ancient DNA.

Ancient DNA from herbarium samples is not directly used in DNA sequencing. This is because over time ancient DNA samples experience spontaneous hydrolytic deamination of cytosine to uracil (Hofreiter et al., 2001). The deamination is often observed in the single-stranded overhangs of a few bases of ancient DNA. During library construction for DNA sequencing, a PCR amplification is applied, which could result in the DNA polymerase substituting thymine for uracil, generating an artificial change of cytosine to thymine (Briggs et al., 2007). To minimize this base misincorporation, a DNA repair protocol with uracil-DNA-glycosylase and endonuclease VIII is applied to the sample before sequencing. First, T4 polynucleotide kinase acts to phosphorylate 5′-phosphate groups of ancient DNA. Next, uracil-DNA-glycosylase replaces uracil residues with abasic sites, and then endonuclease VIII cleaves immediately 5′ and 3′ of the abasic sites, cleaving only the affected strand. Finally, treatment with a T4 polymerase fills in the 5′-overhangs and digests the 3′-overhangs, generating blunt ends that are amenable to Illumina adapter ligation. This DNA treatment is adequate for massively parallel sequencing techniques and reduces the likelihood of base misincorporation (Briggs et al., 2010; Yoshida et al., 2013). This DNA repair protocol is widely applicable to ancient DNA from herbarium specimens.

## Ancient DNA from Infected Plant Tissue is a Mixture of Host and Pathogen but may also Include Other Microbes

The nucleotide sequences obtained from 19th century *P. infestans*-infected potato leaves contained DNA of *P. infestans* and potato but also several species of bacteria (Martin et al., 2013; Yoshida et al., 2013). Alignment of short reads to a reference genome of the target organism enables the exclusion of short reads from untargeted and unrelated organisms from the analysis, but a reference genome may not be available in all cases. In the studies of 19th century *P. infestans*-infected potato leaves, DNA sequences of *P. infestans* were extracted from mixed DNA using the *P. infestans* T30-4 genome sequence (Haas et al., 2009) as reference (Martin et al., 2013; Yoshida et al., 2013). However, one challenge when working with herbarium samples that is also a universal problem when extracting DNA from living infected plant material, is that the percentage of pathogen DNA is highly dependent on the extent of infection or the state of preservation of samples. In the two studies of the 19th century infected potato leaves, 1–20% of DNA was from *P. infestans* (Martin et al., 2013; Yoshida et al., 2013). Therefore, to obtain sufficient coverage of the pathogen genome, deep sequencing must be performed. Another difficulty is the reconstruction of nucleotide sequences from genes or genomic regions that are specific to the historic pathogen. *De novo* assembly of unmapped reads after removing reads that map to the reference genomes of the pathogen, host plant, and other microbes, may allow genome sequence contigs to be identified that could be specific to the historic pathogen. However, to determine whether assembled sequence contigs are from the historic pathogen, the only criteria are those based on homology with DNA sequences of corresponding modern pathogen species.

## Missing Genes in Pathogen DNA from Herbarium Specimens

Ancient pathogen DNA from herbarium specimens is subject to damage and decomposition. In addition, the GC content may influence read coverage across the genome, although this is dependent on the polymerase used in the library construction. If missing genes are estimated based on breadth of read coverage, it is necessary to confirm whether the missing genes are truly biologically lacking in the historic pathogens. The two studies of 19th century *P. infestans* tried to address this when comparing ancient isolates to modern ones (Martin et al., 2013; Yoshida et al., 2013). In cases where a gene deletion was also observed in modern isolates, then the missing gene can be expected to be absent in the historic isolates – particularly if the range of the deleted region in the historic isolate is consistent with that of the modern isolates (Martin et al., 2013).

In cases of genes that appear uniquely absent in the historic pathogen, investigating the genome architecture may help determine the robustness of the observation. For example, the peculiar “two-speed” genome architecture of *P. infestans* could be useful in investigating this issue further. This pathogen has a mosaic genome with two types of regions that evolve at different rates: gene-sparse regions (GSRs) and gene-dense regions (GDRs) (Haas et al., 2009; Raffaele et al., 2010). In the GSRs, effector genes and repeats are highly enriched. GSRs are also enriched in rapidly evolving genes with signatures of positive selection and copy number variation (Raffaele et al., 2010). On the other hand, in the GDRs, repeats are fewer and there are more genes that are conserved among sister species of *P. infestans*. For genes that are absent in the historic samples but present in modern isolates and in the sister species of *P. infestans*, *P. ipomoea*, and *P. mirabilis*, we compared breadth of coverage of sequence reads over genes in GSRs with that in GDRs in the historic samples (Supplemental Table S1). The ratio of genes having zero coverage in GSRs (0.65%) is higher than that in the GDRs (0.31%). This observation is consistent with the findings from the genome analysis of the modern strains that presence/absence polymorphisms of genes more frequently occur in the GSRs than in the GDRs (Raffaele et al., 2010; Cooke et al., 2012). We conclude that most of the missing genes in the herbarium samples could be explained by deletions in the historic strains rather than random decomposition of DNA in the samples.

## Genome Ploidy of Pathogens Preserved in Herbarium Specimens

Plant pathogens have variations in ploidy within and between species. For example, the ploidy level of *Botrityis cinerea* and *P. infestans* is variable within the species (Büttner et al., 1994; Daggett et al., 1995; Catal et al., 2010). The inference of the ploidy level in the historic samples may offer a hint at how historic pathogens have adapted to their host plants. Short-read DNA sequencing can be used to determine the ploidy level based on frequency of mapped short reads at multi- or biallelic positions in one individual, (Yoshida et al., 2013). The frequency of short reads that are derived from one of the alleles corresponds to the frequency of homologous (or homeologous) chromosomes in the genome of one individual. Observed frequencies can be compared to computational simulations of frequency distributions for diploid, triploid, and tetraploid genomes (Yoshida et al., 2013). A frequency of reads originating from one of the alleles of ∼0.5 indicates a diploid. Based on this approach, a 19th century *P. infestans* was deduced to be diploid (Yoshida et al., 2013). The distribution of the frequency in the historic *P. infestans* showed a normal distribution with an average value 0.5, matching the simulated distribution for a diploid (Yoshida et al., 2013). The ploidy level of the historic pathogen is useful for inferring haplotypes as discussed next.

## Haplotype Reconstruction of Genes of the Historic Pathogen

Here we mainly use the term “haplotype” to describe nucleotide sequences of one allele in a gene locus. Haplotype reconstruction is critical for analyzing genetic linkage and studying the function of genes in diploids and polyploids. However, haplotype reconstruction from sequences of ancient DNA from herbarium samples can be challenging. One existing method for haplotype phasing (haplotype construction) and genotype imputation (estimation of genotypes) requires a large number of markers to be assessed across thousands of samples (Browning and Browning, 2007). This limits their application to a small number of organisms such as humans (HapMap project) and *Arabidopsis* (the 1001 Genomes Project). Another method for haplotype phasing is trio sequencing. Genome sequencing of not only an individual but also its parents can distinguish maternal and paternal alleles at positions of single nucleotide polymorphisms (SNPs), increasing the reliability of the haplotypes and genotypes that were inferred from genome sequences of the unrelated individuals (Chen et al., 2013). Since the parent-offspring relationship of the herbarium samples is unknown, their haplotypes cannot be inferred using trio sequencing. In addition, the application of this method is restricted to sexual pathogens. Sasaki et al. (2013) developed a linkage method to detect SNPs and reconstruct haplotypes from a single diploid individual based on the alignment of short sequencing reads. For this method, a single individual is sufficient to enable haplotype reconstruction. As the algorithm is not reliant on long read sequences, reliable haplotypes could be reconstructed from fragmented DNA, making it suitable for ancient DNA.

**FIGURE 1:**
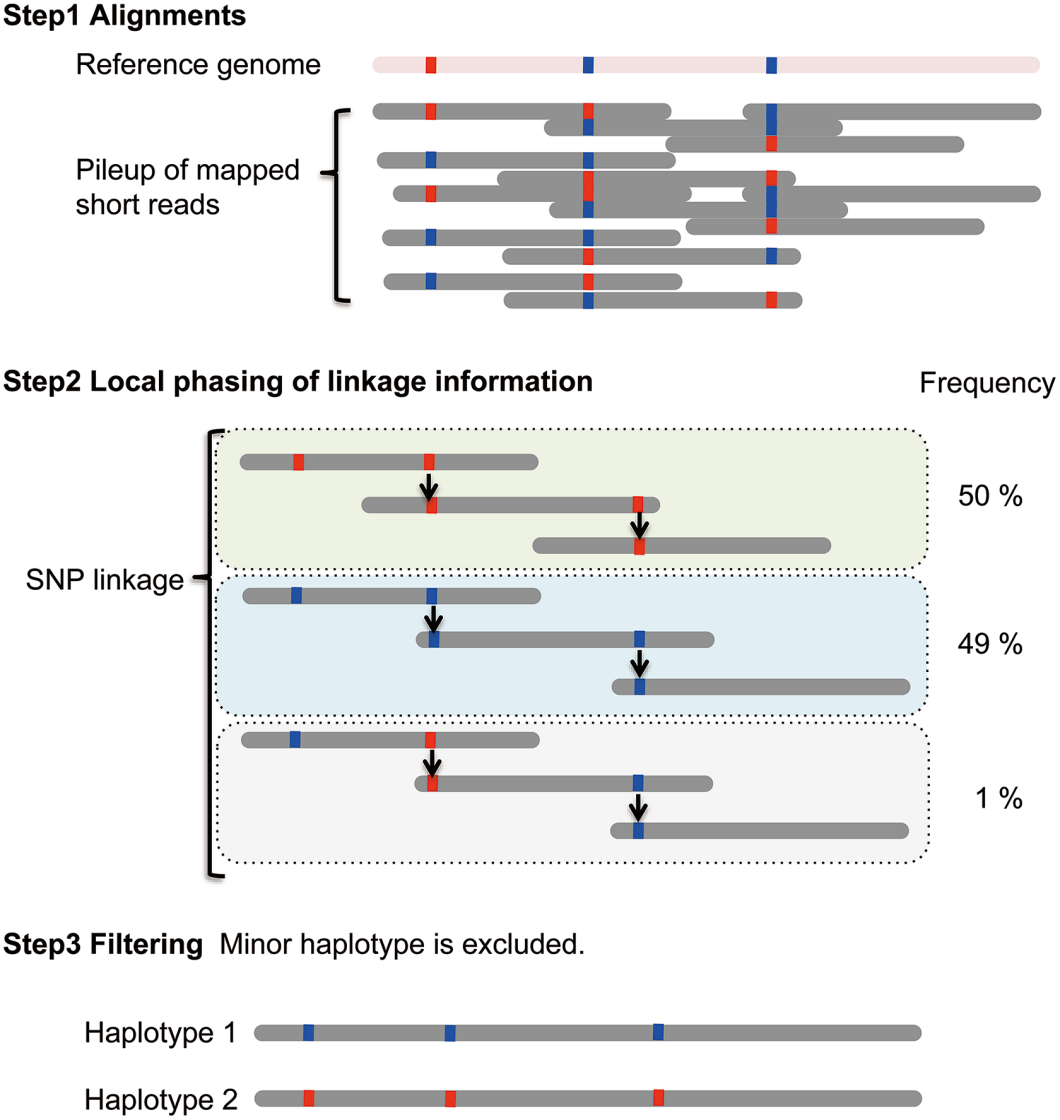
**Local haplotype reconstruction using the linkage method for a single diploid individual**. Scheme was adapted from Sasaki et al. (2013).

In the linkage method, haplotype construction is performed using SNP linkage (**Figure 1**). Briefly, the current diploid algorithm is composed of two parts: local haplotype construction and local haplotype concatenation (Sasaki et al., 2013). In local haplotype construction, short DNA sequencing reads are aligned to the reference genome. Then, the reads are connected using heterozygous SNPs. In **Figure 1**, red and blue boxes indicate different nucleotides at heterozygous SNP positions. In this example, three blocks called local haplotypes are estimated using SNP linkage. Next, minor local haplotypes with low frequency are excluded, resulting in two local haplotypes. This process is performed on partial genomic regions called “windows”. In the second part, the “window” is moved along the chromosome. The local haplotypes obtained can then be assembled into major haplotypes. The local haplotypes are ranked based on scores calculated using their frequency in each of the windows. Based on their rank, local haplotypes are then concatenated. Finally, the two major haplotypes with the highest scores are selected. Ideally, two homologous chromosomes are generated for a diploid organism although in most cases only haplotype blocks are identified, which can range from gene size to much larger regions.

**FIGURE 2:**
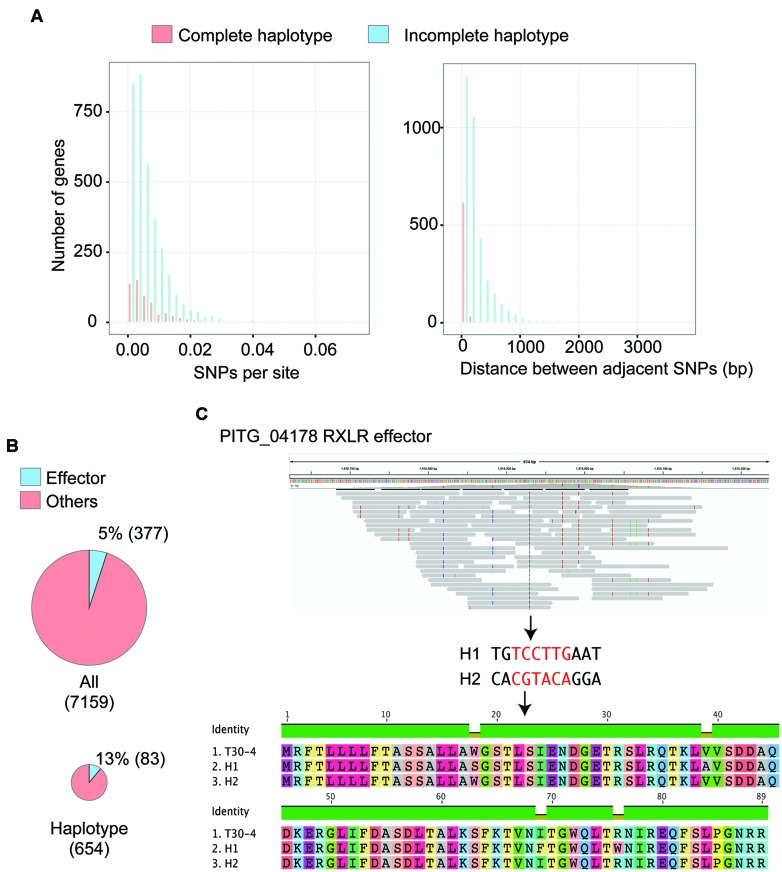
**Haplotype reconstruction of genes in 19th century *Phytophthora infestans* that was preserved in herbarium specimens. (A)** Distributions of single nucleotide polymorphisms (SNPs) per site and physical distance between adjacent SNPs on the genome are shown. Red boxes indicate values for the genes with haplotypes completely reconstructed by linkSNPs software. Blue boxes indicate values for the genes with haplotypes that were not fully recovered. Genes harboring only a single SNP were not included. **(B)** Pie charts showing the number of genes encoding effectors out of the tested genes and the genes with haplotypes that were completely reconstructed. Genes encoding effectors were significantly enriched in the reconstructed haplotypes. **(C)** An example of RXLR effector genes with haplotypes that were successfully rebuilt. PITG_04178 encodes a secreted protein of unknown function containing an RXLR motif. The top image shows the pileup of short reads over the region where PITG_04178 is located using IGV viewer. Colored boxes in the pileup indicate heterozygous SNPs, which were used for haplotype reconstruction. The middle image shows two haplotypes reconstructed by linkSNPs. Red letters indicate nucleotides in the coding region. The bottom image is the alignment of amino acid residues of PITG_04178 that correspond to the modern isolate T30-4 and two haplotypes of 19th century *P. infestans*. One of the haplotypes was consistent with that of T30-4. The other had four amino acid differences.

To test whether this approach is applicable to haplotype reconstruction for individual genes of historic samples that are preserved in herbarium specimens, we used the short reads of *P. infestans* strain HERB-1 (Yoshida et al., 2013). The short reads were merged into single reads and mapped to the *P. infestans* T30-4 reference genome. We only used reads with a mapping quality over 30 (Yoshida et al., 2013). For haplotype construction, we employed linkSNPs software (see Supplemental Material for details), which was developed based on the linkage method (Sasaki et al., 2013). We selected 7,159 genes that showed 100% sequence read coverage over their coding regions and are located in GSRs or GDRs. The software called 56,469 SNPs (44,311 in GDRs and 12,158 in GSRs) and reconstructed 16,702 linkage groups of SNPs, of which 654 allowed deduction of complete haplotypes of the gene (Supplemental Tables S2 and S3). To characterize the differences between genes with complete haplotypes reconstructed and those that were incomplete, we compared the number of SNPs per site and the physical distance of adjacent SNP positions on the genome (**Figure 2A**). We used only genes that had more than one SNP to estimate linkages between SNPs. The successful and unsuccessful cases were similar in the distribution of SNPs per site with an average value of SNPs per site of 0.01 ± 0.01. However, the distance of adjacent SNP positions was significantly different. The average distance between adjacent SNP positions in the genes used to reconstruct the complete haplotype and the incomplete haplotype was 42.8 ± 35.8 bp and 259.5 ± 299.2 bp, respectively. Median length of the ancient DNA in the herbarium specimen was estimated to be 50∼86 bp. Haplotype reconstruction was understandably only applicable to genes with closely linked SNP positions.

Genes with SNP positions that are fairly close may have experienced a high evolutionary rate, a feature of genes encoding effector proteins. In fact, effector genes were significantly enriched among genes with reconstructed haplotypes (**Figure 2B**, Fisher’s exact test, *P* = 1.51e-10). Most effector genes are located in GSRs, reflecting the observation that the rate of successful haplotype reconstruction of genes in GSRs (13.1%) was higher than that in GDRs (7.7%). Among the effector genes with successfully rebuilt haplotypes, 64 effector genes encoded RXLR effector proteins (Supplemental Table S4), a major class of effector genes in *Phytophthora*. For example, the region around gene PITG_04178 encoding an RXLR effector has 11 heterozygous SNPs, six of which are within the coding region of PITG_04178 (**Figure 2C**). The linkSNPs software reconstructed two haplotypes based on these SNPs. One haplotype was consistent with the *P. infestans* T30-4 reference sequence. The other encoded a protein with a four amino acid polymorphism compared to the T30-4 allele.

The linkage method is not applicable to recently duplicated genes. *P. infestans* has another large family of effectors named Crinkler (CRN). Different CRN genes share similar nucleotide sequences due to recent duplications and recombination/shuﬄing of their domains (Haas et al., 2009). Short sequence reads derived from CRN effector genes typically map to multiple locations across the genome reducing mapping quality. We could only rarely reconstruct haplotypes of CRN genes due to the difficulty of obtaining high quality SNPs in these genes. Therefore, the linkage method can be used to reconstruct haplotypes of highly polymorphic genes such as single copy effector genes or anciently duplicated effector genes.

## Conclusion

In conclusion, based on studies of the 19th century *P. infestans*, deep genome sequencing can be applied to obtain sufficient sequence reads from ancient pathogen DNA preserved in herbarium samples. Since *de novo* assembly from fragmented and mixed sequences is difficult, reliable reference genome sequences of the pathogens under study are required. To evaluate missing genes in the historic samples, genome sequencing of multiple samples of both historic and modern pathogens is recommended. The linkage method we described here should be applicable to not only *P. infestans* but also other important pathogens. This enables the accurate reconstruction of allelic and paralogous sequences of effector genes, which could then be synthesized based on the deduced haplotype sequences. Ultimately, this would enable the functional and biochemical characterization of effector proteins that are extinct in the modern biota.

## Conflict of Interest Statement

The authors declare that the research was conducted in the absence of any commercial or financial relationships that could be construed as a potential conflict of interest.
