# Supplementary material for: Computational analyses of ancient pathogen DNA from herbarium samples: challenges and prospects
Source: Front Plant Sci. 2015 Sep 24;6:771. doi: 10.3389/fpls.2015.00771 (PMC4585160; doi:10.3389/fpls.2015.00771)
Supplement: Supplementary file 1 [file Data_Sheet_1.DOCX]

***Supplementary Material***

**Computational analyses of ancient DNA from herbarium samples: challenges and prospects**

**Kentaro Yoshida^1,2^*, Eriko Sasaki^2^, Sophien Kamoun^3^**

^1^ Laboratory of Plant Genetics, Graduate School of Agricultural Science, Kobe University, Kobe, Japan.　　　　　　^2^The Sainsbury Laboratory, Norwich Research Park, Norwich, UK

^3^ Gregor Mendel Institute, Austrian Academy of Sciences, Vienna, Austria

*** Correspondence:** Kentaro Yoshida, Laboratory of Plant Genetics, Graduate School of Agricultural Science, Kobe University, Kobe, 657-0013, Japan.

kentaro.yoshida@port.kobe-u.ac.jp

1. **Supplementary Data**

**1.1 Methods**

Breadth coverage over genes

We used the data (ERP002419) in Yoshida et al. 2013 to compare breadth of coverage of sequence reads over genes in GSRs with that in GDRs in the historic samples. The coverage over genes for the historic samples was estimated by combining alignment files of the 11 historic isolates (M-0182907, M-0182896, KM177517, KM177513, M-0182898, KM177500, KM177514, KM177512, KM177509, KM177548 and M-0182903). The coverage over genes for the modern samples was estimated by combining alignment files of the 7 modern isolates (P6096, P1362, P11633, P10650, P12204, P17721, P10127). In addition, the coverage over genes was estimated based on each of alignment files of another 7 modern isolates (06_3938A, P13527, PI3626, NL07434, P17777, DDR7602, LBUS5), *P. mirabilis* PIC99114 and *P. ipomoea* PIC99167. If the gene showed zero coverage in both modern and historic samples, we did not count the gene as absence in the historic samples. We used only mapped reads with a mapping quality over 30. The genes with zero coverage in both modern and historic samples are potentially multiple copy genes, which were recently duplicated. We regarded a gene as absence in the historic samples if zero coverage was detected only in the historic sample.

Haplotype reconstruction

We used the alignment file of the M-0182896 isolate to reconstruct haplotypes of genes. M-0182896 isolates has the highest coverage of reads over genome in the historic isolates (Yoshida et al. 2013). Using bedtools v2.24.0, we extracted short reads that were mapped to the genomic regions, in which genes are located. We targeted only the genes that showed 100% sequence read coverage over their coding regions and are located in GSRs or GDRs (Supplemental Table 1). To call SNPs and reconstruct haplotypes, we employed linkSNPs ver1.2 beta (Sasaki et al. 2013) with default conditions. linkSNPs software generates files containing SNPs, their positions on the genome, and haplotypes. We used only the genes with more than one SNP for evaluation of haplotype reconstruction. SNPs per site and physical distance between adjacent SNPs were calculated based on the outputs from linkSNPs software (Supplemental Tables 2 and 3).

References

Quinlan, A. R., and Hall, I. M. (2010). BEDTools: A flexible suite of utilities for comparing genomic features. *Bioinformatics* 26, 841–842. doi:10.1093/bioinformatics/btq033.

Sasaki, E., Sugino, R. P., and Innan, H. (2013). The linkage method: A novel approach for SNP detection and haplotype reconstruction from a single diploid individual using next-generation sequence data. *Mol. Biol. Evol.* 30, 2187–2196. doi:10.1093/molbev/mst103.

Yoshida, K., Schuenemann, V. J., Cano, L. M., Pais, M., Mishra, B., Sharma, R., Lanz, C., Martin, F. N., Kamoun, S., Krause, J., et al. (2013). The rise and fall of the Phytophthora infestans lineage that triggered the Irish potato famine. *Elife* 2013, 1–25. doi:10.7554/eLife.00731.

1. **Supplementary Tables**

**Supplementary Table 1. Breadth coverage over genes in 7 modern isolates and 11 historic isolates of *Phytophthora infestans.***

**Supplementary Table 2. The reconstructed haplotypes of genes of the HERB-1 isolate that are located in GDRs**.

**Supplementary Table 3. The reconstructed haplotypes of genes of the HERB-1 isolate that are located in GSRs**.

**Supplementary Table 4. The reconstructed haplotypes of effector genes of the HERB-1 isolate.**
